# Laparoscopic versus open surgery for colonoscopic perforation: A systematic review and meta-analysis

**DOI:** 10.1097/MD.0000000000034057

**Published:** 2023-06-16

**Authors:** Wu Zhong, Chuanyuan Liu, Chuanfa Fang, Lei Zhang, Xianping He, Weiquan Zhu, Xueyun Guan

**Affiliations:** a Department of General Surgery, The Ganzhou People’s Hospital, Ganzhou, China; b Department of Pediatric, The Ganzhou People’s Hospital, Ganzhou, China.

**Keywords:** colonic perforation, colonoscopy, laparoscopic surgery, laparoscopy

## Abstract

The efficacy of laparoscopic surgery (LS) for the treatment of colonoscopic perforation is still controversial. The purpose of this meta-analysis was to evaluate the effectiveness and safety of LS versus open surgery (OS) for colonoscopic perforation.

**Methods:**

All clinical trials that compared laparoscopic with OS for colonoscopic perforation published in English were identified in PubMed, EMBASE, Web of Science, and Cochrane Library searches. A modified scale was used to assess the quality of the literature. We analyzed the age, sex ratio, aim of colonoscopy, history of abdominopelvic surgery, type of procedure, size of perforation, operation time, postoperative fasting time, hospital stay, postoperative complication morbidity, and postoperative mortality. Meta-analyses were performed using weighted mean differences for continuous variables, and odds ratios for dichotomous variables.

**Results:**

No eligible randomized trials were identified, but eleven nonrandomized trials were analyzed. In the pooled data of 192 patients who underwent LS and 131 OS, there were no significant differences in age, sex ratio, aim of colonoscopy, history of abdominopelvic surgery, perforation size, and operative time between the groups. LS group had shorter time of hospital stay and postoperative fasting time, less postoperative complication morbidity, but there were no significant difference in postoperative mortality rate between LS group and OS group.

**Conclusions:**

Based on the current meta-analysis, we conclude that LS is a safe and efficacious technique for colonoscopic perforation, with fewer postoperative complications, less hospital mortality, and faster recovery compared with OS.

## 1. Introduction

The frequency of perforations from colonoscopy is estimated to be 0.016 to 0.8% for diagnostic colonoscopy and 0.02 to 8% for therapeutic colonoscopy.^[1,2]^ Because of the widespread application of endoscopic submucosal dissection for the past few years, the incidence of perforation after colonoscopy were increasing rapidly.^[3]^ If not properly handled, it can lead to peritonitis, sepsis, and even death. Treatment must be tailored according to the patient’s comorbidities and clinical status as well as the specific conditions during the colonoscopy that led to the perforation.^[4]^ The managements of colonic perforations included operative and nonoperative methods. There were several researches supported nonoperative management in patients with no evidence of peritonitis and good clinical condition.^[5,6]^ Nevertheless, nonoperative management has the possibility of failure, and surgical procedures might be inevitable if the condition continue getting worse. Some studies have reported endoscopic clip closure for the treatment of iatrogenic colon perforations, but there is still some possibility of failure.^[7,8]^ By far, operation is recognized as the most reliable treatment for colonic perforation.^[9,10]^

Previously, most surgeons chose open surgery (OS) for colonic perforation, but it had disadvantages such as greater trauma and slower recover. As the use of laparoscopic techniques in colorectal surgery has been widely accepted, it has become a better choice for the treatment of colonoscopic perforation.^[11]^ In recent years, there were a few researches that have assessed the efficacy of laparoscopic surgery (LS) in the treatment of colonoscopic perforation. The advantages are evident, LS has the same therapeutic outcomes, as well as less pain, shorter hospital stay, and less perioperative morbidity, compared with traditional OS.^[12–14]^ In general, these have been limited to case reports and single-center studies, the efficacy of LS for the treatment of colonoscopic perforation is still controversial. Therefore, we carried out this meta-analysis, which could be used to help surgeons in choosing a better approach for the management of colonic perforation during colonoscopy.

## 2. Materials and methods

### 2.1. Literature search

We identified studies by searches of electronic databases such as PubMed, EMBASE, Web of Science, and Cochrane Library. The keywords used for the search included “colon perforation,” “colonic perforation,” “colonoscopic perforation,” “colonoscopy,” “laparoscopic,” and “laparoscopy.” The latest search was updated on March 30, 2022. Reference lists from identified publications were also reviewed so as to search for potentially relevant studies.

### 2.2. Inclusion and exclusion criteria

Two reviewers (ZW and LCY) evaluated every retrieved study independently. Inclusion criteria were as follows: studies that made a comparison between LS and OS for the treatment of colonic perforation during colonoscopy; recorded the majority of the following: aim of colonoscopy, history of abdominopelvic surgery, perforation size, operative time, postoperative fasting time, hospital stay, postoperative complication, postoperative mortality, blood loss, types of antibiotics used after surgery and readmission rate; were written in English; if the studies were from the same population, the most informative and recent study were included. Exclusion criteria included: did not use OS as a control; duplicate publication or the publication provided insufficient data; abstracts, letters, case reports, and reviews.

### 2.3. Data extraction and quality assessment

Data from all the eligible studies were extracted and checked by 2 independent investigators (ZW and LCY). Disagreements in data extraction were resolved by discussion with other members (FCF) in our group. The following information were extracted from each study: the publication year, study period, the number of participants, mean age, sex ratio, aim of colonoscopy, history of abdominopelvic surgery, type of procedure, size of perforation, operation time, postoperative fasting time, hospital stay, postoperative complication morbidity, and postoperative mortality. If the study provided medians/ranges instead of means/standard deviations (SDs), we transformed the medians/ranges to mean/SDs using the technique described by Hozo.^[15]^ A modified scale method was used to assess the quality of literature according to a previously established scoring system, the methodological index for nonrandomized studies (Table 1).^[16]^

**Table 1:** Modified MINORS scale used for quality assessment of nonrandomized controlled trials.

Item	Points		
	0	1	2
Contemporary groups	Not reported	Study group compared with historical control group	Study group compared with contemporary control group
Prospective collection of data	Not reported	Data obtained from retrospective review of medical history	Data obtained from prospectively maintained database
Inclusion of consecutive patients	Not reported	Patients are not consecutive	Patients are consecutive
Baseline equivalence of groups	No matching analysis performed	Matching incomplete	Matching complete
A control group having the gold standard intervention	Not reported	Incomplete report of the standard intervention	Complete report of the standard intervention
Important data being presented	Lack	Less comprehensive	comprehensive
Sample size of LS group	<20	20–50	>50

LS = laparoscopic surgery, MINORS = methodological index for nonrandomized studies.

### 2.4. Statistical analysis

Meta-analysis was performed using Review Manager Version 5.2 software (The Cochrane Collaboration, Oxford, UK). Weighted mean differences (WMD) with 95% confidence intervals (CIs) were used for analyzing continuous variables and odds ratios (ORs) for dichotomous variables. Heterogeneity was assessed by using the Cochran *Q* test and *I*^2^ statistics. If the *P* < .1 and *I*^2^ exceeded 50%, heterogeneity was considered to indicate statistical significance.^[17]^ Random effects model was used to identify heterogeneity among the studies, otherwise, fixed-effects model was used. Potential sources of heterogeneity were explored by carrying out subgroup analyses. Sensitivity analysis was performed by eliminating each study at a time from the meta-analysis. Funnel plots were used to assess the potential publication bias.

### 2.5. Ethics and dissemination

Our aim is that this systematic review could be published in a peer-reviewed journal. The results will evaluate the effectiveness and safety of LS versus OS for colonoscopic perforation. Participants’ privacy not being involved, this systematic review will not require informed consent form.

## 3. Results

### 3.1. Selected studies

**Figure 1.**
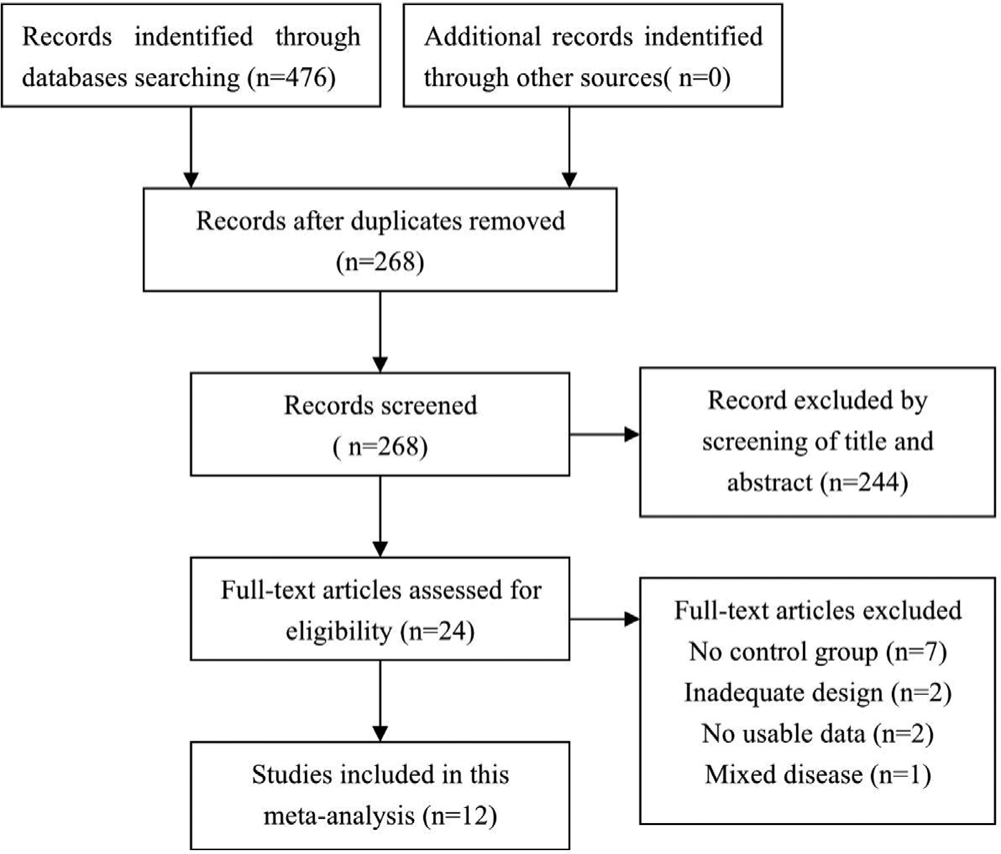
Literature search and selection procedures.

A total of 476 potential articles were retrieved according to the initial search strategy. The titles and abstracts of 268 articles were reviewed after excluding duplicated articles. Two hundred forty-four articles were excluded by this process, and the full text of 24 articles was reviewed. Of these, 12 articles were excluded for the following reasons: no control group (n = 7); inadequate design (n = 2); no usable data (n = 2); mixed disease (n = 1). Ultimately, 11 articles^[12,13,18–26]^ (America = 2, Argentina = 1, Belgium = 1, Germany = 2, Korea = 3, China = 2) totaling 323 patients were finally eligible for the quality assessment,of whom 192 underwent LS and 131 underwent OS. A flowchart of the literature search and selection is illustrated in (Fig. 1).

### 3.2. Study characteristics and quality assessment

These included 11 non-randomized clinical trials^[12,13,18–26]^ (NRCTs) totaling 323 patients published between 2007 and 2020. The sample size of these studies ranged from 11 to 99. In these retrospective and nonrandomized studies, a true comparison of postoperative outcomes between open and LS cannot be performed because the 2 groups of patients are not identical. For the nature of the laparoscopy surgery trials, it was also impossible to perform blinding. The diagnosis of colon perforation are dependent on direct visualization during colonoscopy, abdominal plain radiography, or computed tomography of abdomen. The surgical management of colonoscopic perforation included primary repair or bowel resection, with or without proximal stoma diversion. The characteristics of the studies included in this meta-analysis are presented in Table 2. The quality assessment of the NRCTs is shown in Table 3.

**Table 2:** Characteristics of included studies comparing LS with OS for colonoscopic perforation.

Study	Study period	Country	Number of patients		Sex of patients (M/F)		Aim of colonoscopy (diagnostic/therapeutic)		History of abdominopelvic surgery (yes/no)		Type of procedure
			LS	OS	LS	OS	LS	OS	LS	OS	
Hansen AJ^[18]^	2007	America	7	4	3/4	1/3	6/1	1/3	7/0	2/2	Primary repair, stapled repair, colostomy
Bleier JI^[19]^	2008	America	11	7	2/9	3/4	NA	NA	NA	NA	Primary repair
Rumstadt B^[21]^	2008	Germany	10	3	NA	NA	NA	NA	NA	NA	Primary repair
Rotholtz NA^[20]^	2010	Argentina	14	6	5/9	2/4	8/6	3/3	NA	NA	Primary repair, colonic resection, Hartmann, colostomy
Coimbra C^[22]^	2011	Belgium	16	23	9/7	11/12	11/5	17/6	3/13	7/16	Primary repair, colostomy
Schlöricke E^[23]^	2013	Germany	24	12	14/10	5/7	NA	NA	4/20	4/8	Primary repair, colonic resection
Kim J^[24]^	2014	Korea	17	8	8/9	4/4	NA	NA	NA	NA	Primary repair, colonic resection, Hartmann
Zhong W^[26]^	2016	China	13	8	7/6	5/3	NA	NA	0/13	0/8	Primary repair,
Shin DK^[25]^	2016	Korea	8	15	7/1	5/10	4/4	7/8	0/8	3/12	Primary repair, colonic resection, colostomy
Lee JS^[12]^	2020	Korea	59	40	35/24	22/18	31/28	22/18	11/48	8/32	Primary repair, colonic resection, Hartmann, colostomy
Li L^[13]^	2020	China	13	5	7/6	2/3	8/5	5/0	NA	NA	Primary repair, colostomy

LS = laparoscopic surgery, OS = open surgery.

**Table 3:** Modified MINORS score of all eligible nonrandomized comparative studies.

First author	Year	Contemporary groups	Prospective data collection	Consecutive patients	Baseline equivalent of groups	Gold standard intervention	Important data being presented	LS sample size	Score
Hansen AJ	2007	2	1	2	0	2	2	0	9
Bleier JI	2008	2	1	2	2	2	1	0	10
Rumstadt B	2008	2	1	2	2	2	0	0	9
Rotholtz NA	2010	2	1	2	2	2	0	0	9
Coimbra C	2011	2	1	2	2	2	2	0	11
Schlöricke E	2013	2	1	2	2	2	2	1	11
Kim J	2014	2	1	2	2	2	2	0	11
Zhong W	2016	2	1	2	2	2	2	0	11
Shin DK	2016	2	1	2	2	2	2	0	11
Lee JS	2020	2	1	2	2	2	2	2	13
Li L	2020	2	1	2	2	2	2	0	11

LS = laparoscopic surgery, MINORS = methodological index for nonrandomized studies.

## 4. Meta-analysis results

### 4.1. Age

**Figure 2.**
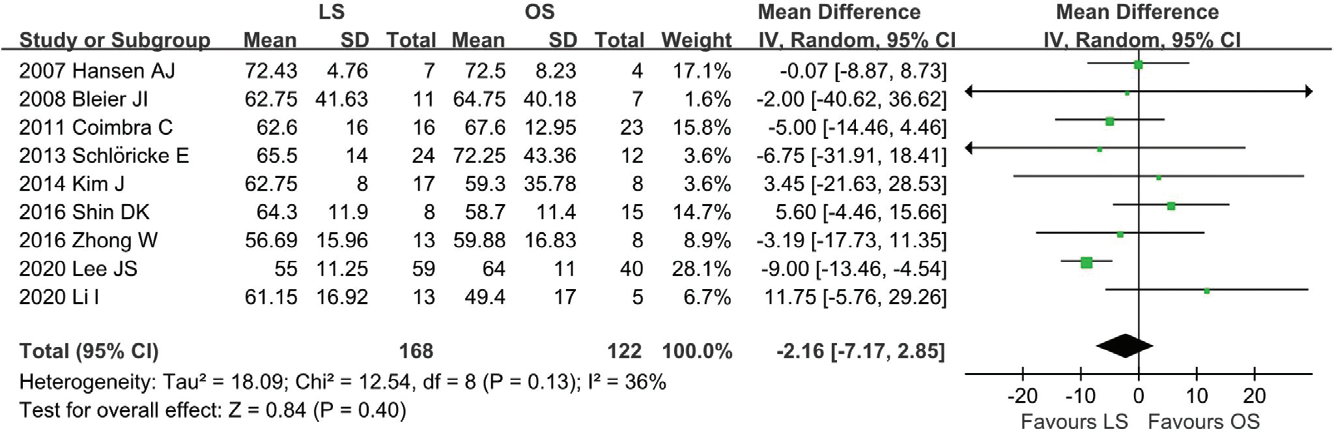
Meta-analysis of age.

A total of 9 studies^[12,13,18,19,22–26]^ were included (LS = 168, OS = 122) and no statistical heterogeneity was identified among these studies (*I*^2^ = 36%, *P* = .13). In the pooled data, there was no significant difference in the age between the groups (WMD: −2.16; 95% CI: −7.17 to 2.85; *P* = .40; Fig. 2).

### 4.2. Sex ratio

**Figure 3.**
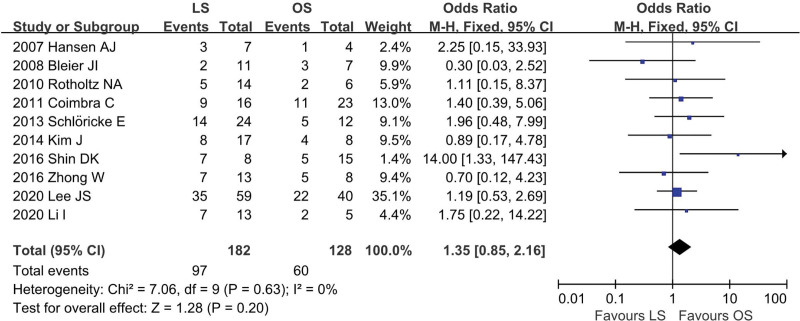
Meta-analysis of sex ratio.

Ten studies^[12,13,18–20,22–26]^ were included (LS = 182, OS = 128), and low heterogeneity among the studies (*I*^2^ = 0%, *P* = .63). In the pooled data, there was no significant difference in sex ratio between the groups (OR = 1.35; 95% CI: 0.85 to 2.16; *P* = .20; Fig. 3).

### 4.3. Aim of colonoscopy

**Figure 4.**
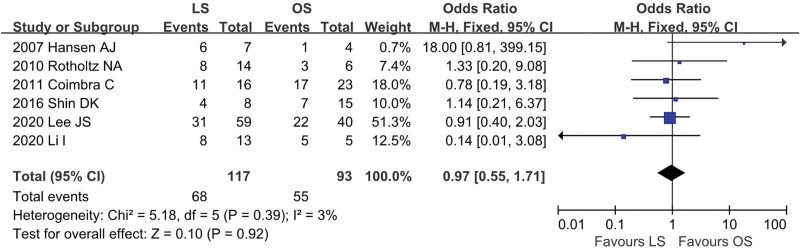
Meta-analysis of aim of colonoscopy.

Six studies^[12,13,18,20,22,25]^ provided data regarding the aim of colonoscopy (LS = 117, OS = 93). There was low heterogeneity among the studies (*I*^2^ = 3%, *P* = .39). In the pooled data, there was no significant difference in the aim of colonoscopy between the groups (OR = 0.97; 95% CI: 0.55 to 1.71; *P* = .92; Fig. 4).

### 4.4. History of abdominopelvic surgery

**Figure 5.**
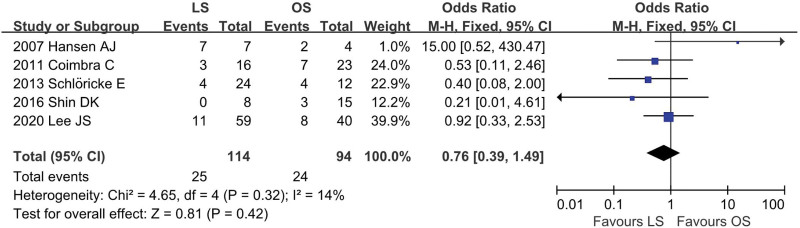
Meta-analysis of history of abdominopelvic surgery.

Five studies^[12,18,22,23,25]^ provided data regarding the history of abdominopelvic surgery (LS = 114, OS = 94). There was low heterogeneity among the studies (*I*^2^ = 14%, *P* = .32). In the pooled data, there was no significant difference in the history of abdominopelvic surgery between the groups (OR = 0.76; 95% CI: 0.39 to 1.49; *P* = .42; Fig. 5).

### 4.5. Perforation size

**Figure 6.**
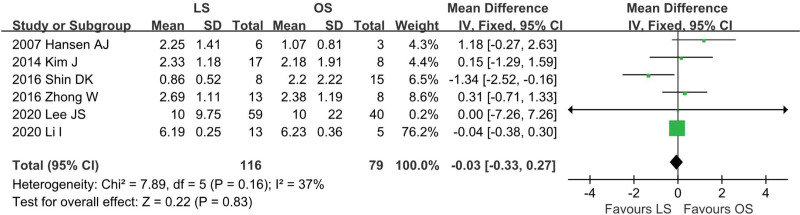
Meta-analysis of perforation size.

Six studies^[12,13,18,24–26]^ provided data regarding perforation size (LS = 116, OS = 79). There was low heterogeneity among the studies (*I*^2^ = 37%, *P* = .16). There was no significant difference in the pooled data (WMD = −0.03; 95% CI: −0.33 to 0.27; *P* = .83; Fig. 6).

### 4.6. Operative time

**Figure 7.**
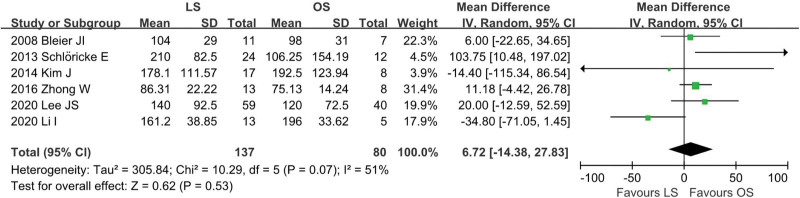
Meta-analysis of operative time.

Data on operation time was available from 6 studies^[12,13,19,23,24,26]^ (LS = 137, OS = 80). There was moderate heterogeneity among the studies (*I*^2^ = 51%, *P* = .07). In the pooled data, there was no significant difference in operation time between the groups (WMD = 6.72, 95% CI: −14.38 to 27.83; *P* = .53; Fig. 7).

### 4.7. Postoperative fasting time

**Figure 8.**
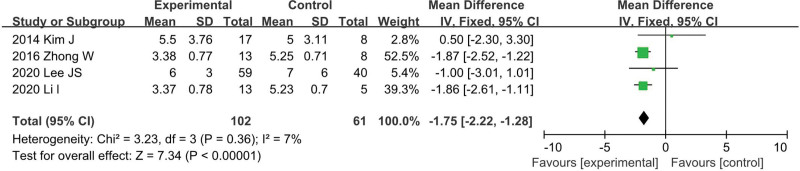
Meta-analysis of postoperative fasting time.

Data on operation time was available from 4 studies^[12,13,24,26]^ (LS = 102, OS = 61). There was no heterogeneity among the studies (*I*^2^ = 7%, *P* = .36). In the pooled data, LS group was associated with significantly decreased postoperative fasting time among these studies (WMD = −1.75; 95% CI: −2.32 to −1.28; *P* < .001; Fig. 8).

### 4.8. Hospital stay

**Figure 9.**
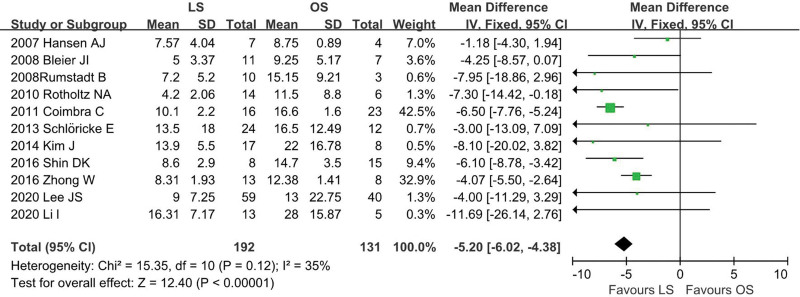
Meta-analysis of hospital stay.

Eleven studies^[12,13,18–26]^ provided data regarding duration of postoperative hospital stay (LS = 192, OS = 131). In the pooled data, LS group was associated with significantly decreased hospital stay (WMD = −5.20; 95% CI: −6.02 to −4.38; *P* < .001) with low heterogeneity among these studies (*I*^2^ = 35%; *P* = .12; Fig. 9).

### 4.9. Postoperative complication morbidity

**Figure 10.**
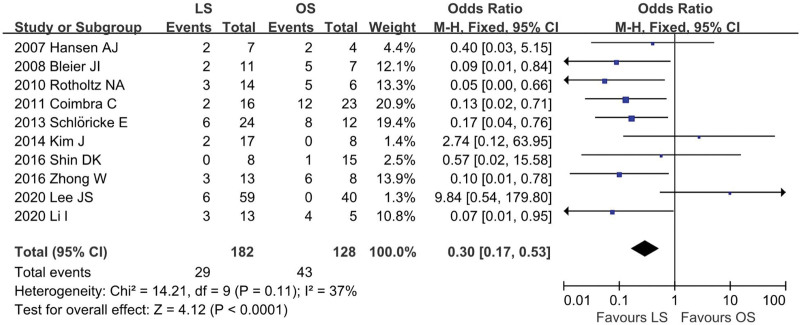
Meta-analysis of postoperative complication morbidity.

Ten researches^[12,13,18–20,22–26]^ provided data regarding postoperative complications (LS = 182, OS = 128). The combined result indicated that the overall postoperative complication morbidity was significantly less in the LS group than in the OS group (OR = 0.30; 95% CI: 0.17 to 0.53; *P* < .001). The overall analysis in the studies show low heterogeneity (*I*^2^ = 37%; *P* = .11; Fig. 10). The complications include in this meta-analysis were ileus, wound infection, fever, abscess, pneumonia, anastomotic leak, and so on.

### 4.10. Postoperative mortality

**Figure 11.**
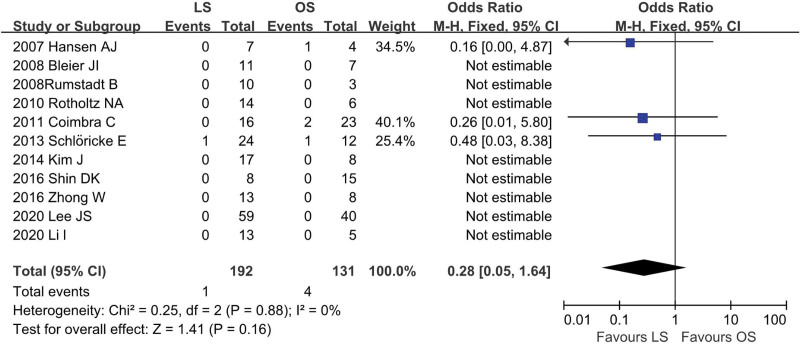
Meta-analysis of postoperative mortality.

The postoperative mortality rates were reported in 11 studies^[12,13,18–26]^ (LS = 192, OS = 131). Meta-analysis demonstrated that no significant difference in postoperative mortality rate between LS group and OS group (OR = 0.28; 95% CI: 0.05 to 1.61; *P* = .16), with no significant heterogeneity among studies (*I*^2^ = 0%; *P* = .88; Fig. 11).

### 4.11. Sensitivity analysis and publication bias

**Figure 12.**
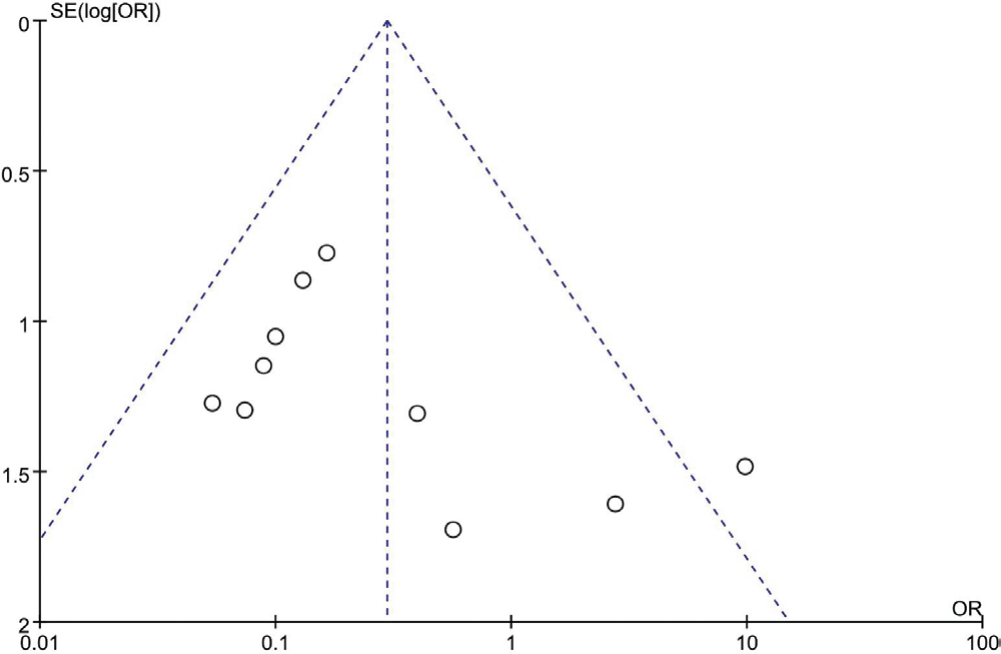
Funnel plots of postoperative complication morbidity.

Analysis of sensitivity was made by removing each study at a time from the meta-analysis, the results have not altered. Publication bias was assessed for each complication by using the funnel plot of the included studies, visual inspection of the funnel plot revealed symmetry, suggesting no severe publication bias (Fig. 12).

## 5. Discussion

LS is a technique that attracts an increasing number of surgeons, thanks to its offering important advantages (less pain, shorter hospital stay, and less perioperative morbidity) over OS for patients with colorectal diseases.^[27,28]^ However, the efficacy of LS for the treatment of colonoscopic perforation is still controversial. Our meta-analysis included 11 nonrandomized controlled studies^[12,13,18–26]^ that compared the short-term outcomes of LS with OS for the treatment of colonoscopic perforation. The modified methodological index for nonrandomized studies scale method was used to assess the quality of these NRCTs and the score of included studies was no less than 8. In this meta-analysis, we focused on not only the short-term outcomes but also including age, sex ratio, aim of colonoscopy, history of abdominopelvic surgery and perforation size. These subsequent 5 items were used to assess whether there had been selection bias in enrollment for the studies analyzed. Our meta-analysis revealed that there were no significant differences in age, sex ratio, aim of colonoscopy, history of abdominopelvic surgery, and perforation size between LS group and OS group. This demonstrated that the patients included in our studies were comparable between the groups.

Some researches reported that LS remains a time-consuming procedure even conducted by experienced surgeons.^[29,30]^ Our meta-analysis revealed that there was no significant difference in operation time between the groups. There are several reasons as follows: first, a large proportion of patients included in our studies underwent laparoscopic primary repair, rather than bowel resection; second, it is well known that LS could enlarge the surgical field, which would permit better exposure and allow better identification of the site of perforation;^[31]^ Third, during the past 20 years, equipment of laparoscopy was more advanced and the surgeons more and more skilled laparoscopic techniques. Thus, we believe that with the continued development of endoscopic equipment and laparoscopic technique, the operation time required for LS will become shorter in the future.^[32,33]^

Many studies have shown that LS has the advantages of less trauma and recover quickly.^[33]^ Our meta-analysis results indicated that the time of postoperative fasting and hospital stay were shorter for the LS group than the OS group. It means that patients who underwent LS recovered more quickly than those who underwent OS. This is easy to understand, LS has a shorter incision than OS, resulting in less postoperative pain, and patients are more receptive to early ambulation, which improves gastrointestinal function and allows them to eat food earlier than the open group. Shorter postoperative fasting time can speed up the patient’s recovery process, leading to shorter hospital stays. In this meta-analysis, data from the 11 studies included showed that the length of hospital stay was shorter in the LS group than in the OS group. These strongly prove that LS is superior to OS in terms of postoperative recovery.

The incidence of surgical complications and mortality is critical for a surgical technique. Our meta-analysis revealed that postoperative complication morbidity after LS was fewer than OS. This finding might have been due to the minimal trauma of LS. LS has a smaller incision, and if the abdominal cavity is contaminated, the incision can be isolated by trocar without contamination, so the probability of infection of the incision is less than that of OS. Because patients have smaller incisions, less pain, and move out of bed earlier, complications such as pneumonia and fever were also reduced. Among the 10 studies included in the meta-analysis, only Kim’s study showed a higher incidence of postoperative complications. But interestingly, the length of hospital stay in the LS group was still shorter than that in the OS group.

One and 4 postoperative cases of death in LS group and OS group, respectively. No mortality occurred during postoperative period was reported in 8 studies.^[12,13,19–21,24–26]^ In the pooled data, there was no significant difference in postoperative mortality rates between the groups but fewer deaths occurred in the LS group than in the OS group. This result strongly suggested that LS is an effective treatment and could achieve a comparable short-term prognosis for colonoscopic perforation compared with OS group.

This is the first meta-analysis study to compare the short-term outcomes between OS and LS for colonoscopic perforation. We fully acknowledge that the low number of patients in our study and the included studies were all NRCTs. The findings of high-quality NRCTs might be as reliable as randomized controlled trials, particularly when pooled data are compared to evaluate the effectiveness of surgical procedures.^[34]^ Considering the low incidence of colonic perforation during colonoscopy, we think our research is still valuable.

Nonetheless, our study actually owns some limitations: first, all the included studies are NRCTs, which may exaggerate the effect magnitude of an intervention. Second, heterogeneity was detected within several outcomes, specifically perforation size and operative time. The degree of between-study heterogeneity present may undermine the quality and legitimacy of the results obtained, even though we already used the random model.^[35]^ Third, the number of enrolled patients in all the included studies is relatively small, which is likely to have masked the true difference in some variables. Fourth, transforming abnormal distribution (media/range) to normal distribution (mean/SD) is likely to subject to a potential bias. Fifth, all the surgical outcomes might be influenced by the surgeon’s experience. However, most studies did not explicitly state whether surgeons were proficient in the LS technique before the trial started. Finally, although an extensive literature search was done, we may miss some unpublished studies.

## 6. Conclusion

Based on the current meta-analysis, LS is a safe and efficacious technique for colonoscopic perforation, with fewer postoperative complications, less hospital mortality, and faster recovery compared with OS. However, this does not mean that LS is an alternative method to OS. We maintain whether LS should be implemented based on the experience of a laparoscopist, and several factors should be taken into account, such as good medical condition, history of abdominopelvic surgery, small size of perforation, and clean bowel preparation.^[19]^ When a surgeon feels that it is difficult to complete by the laparoscopic method, conversion to an open procedure is always a consideration.

## Author contributions

**Conceptualization:** Xueyun Guan.

Data curation: Wu Zhong.

Investigation: Wu Zhong, Chuanyuan Liu.

Methodology: Chuanfa Fang, Xianping He.

Writing – original draft: Wu Zhong, Lei Zhang, Weiquan Zhu.
